# Supplementary material for: Successful Spermatogonial Stem Cells Transplantation within Pleuronectiformes: First Breakthrough at inter-family Level in Marine Fish
Source: Int J Biol Sci. 2021 Oct 25;17(15):4426–41. doi: 10.7150/ijbs.63266 (PMC8579436; doi:10.7150/ijbs.63266)
Supplement: Supplementary file 1 — Supplementary figure. [file ijbsv17p4426s1.pdf]

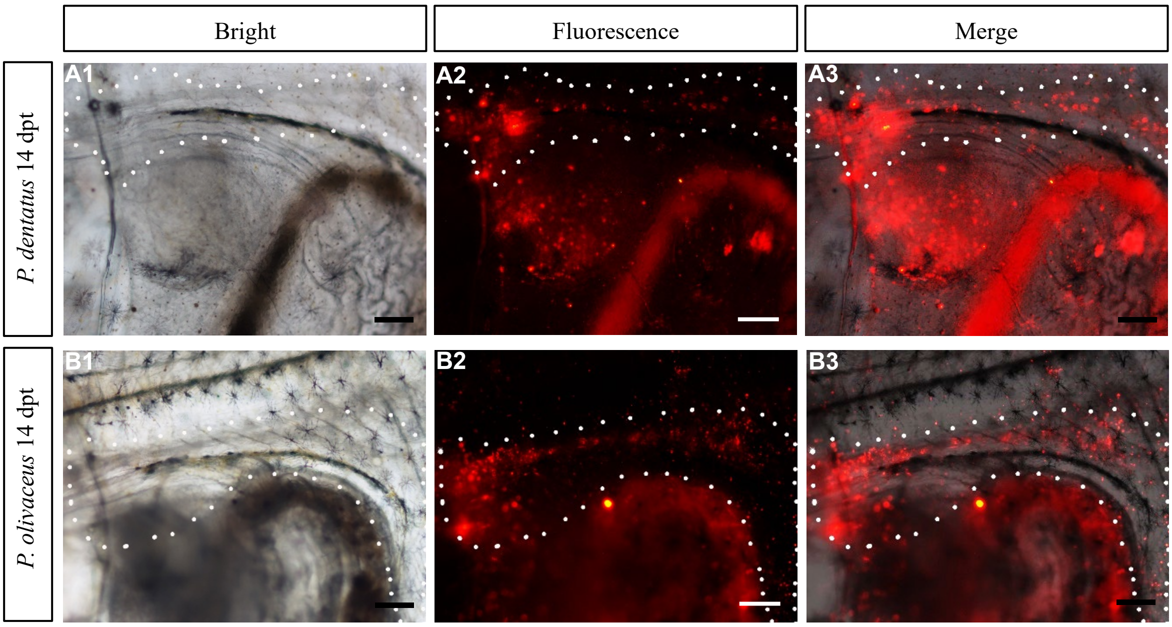


**Figure S1. Assessment the migration and colonization rate of transplanted donor cells in recipients.** (A1-A3) Observation PKH26-labeled cells of summer flounder at genital ridge of 14 dpt recipients. (B1-B3) Observation PKH26-labeled cells of Japanese flounder at genital ridge of 14 dpt recipients. Scale bar, 100 μm.
